# Primary Somatosensory to Motor Cortex Microstructural Connectivity Predicts the Mu‐Rhythm Phase Effect on Corticospinal Excitability: An EEG‐TMS Study

**DOI:** 10.1002/hbm.70576

**Published:** 2026-06-04

**Authors:** Juliana R. Hougland, Timo Roine, Andreas Jooß, Dania Humaidan, Ulf Ziemann

**Affiliations:** ^1^ Department of Neurology & Stroke University of Tübingen Tübingen Germany; ^2^ Hertie Institute for Clinical Brain Research Tübingen Germany; ^3^ Deparment of Neuroscience and Biomedical Engineering Aalto University School of Science Espoo Finland; ^4^ School of Smart Solutions Metropolia University of Applied Sciences Helsinki Finland

**Keywords:** corticospinal excitability, diffusion MRI, EEG‐TMS, MEP, mu‐rhythm, phase, sensorimotor cortex

## Abstract

The sensorimotor mu‐rhythm phase modulates corticospinal excitability, but mu‐phase effects on motor evoked potentials (MEPs) elicited with transcranial magnetic stimulation (TMS) vary substantially across individuals. Structural connectivity between primary motor cortex (M1) and primary somatosensory cortex (S1) may explain variability in effect strength. We examined whether S1–M1 microstructural connectivity relates to mu‐phase effect strength on MEP amplitudes. A total of 28 individuals completed diffusion magnetic resonance imaging (dMRI) and electroencephalography (EEG)–TMS. Fiber tractography between S1 and M1 was performed. Quantitative anisotropy and diffusion‐tensor imaging metrics including fractional anisotropy, radial diffusivity, and axial diffusivity were extracted from S1–M1 fibers, along with neurite orientation dispersion and density imaging metrics. Single‐pulse TMS was delivered to left M1. Mu‐phase was calculated from pre‐stimulus EEG, and MEP amplitudes were calculated from right‐hand muscles. Mu‐phase effect strength was quantified using a circular‐to‐linear correlation of mu‐phase and MEP amplitude. Spearman correlations assessed relationships between the dMRI measures and mu‐phase effect strength. Whole‐brain correlational tractography was performed to explore other tracts associated with mu‐phase effect strength. Higher S1–M1 quantitative anisotropy was associated with stronger mu‐phase effect on MEP amplitudes in both hemispheres (left: *ρ* = 0.384, *p* = 0.044; right: *ρ* = 0.409, *p* = 0.032). Exploratory whole‐brain correlations identified additional tracts relevant to mu‐phase effect strength. In conclusion, S1–M1 microstructural connectivity influences mu‐phase modulation of corticospinal excitability. Communication between S1–M1 is important for effective phase‐dependent TMS. dMRI could be used to predict individuals' potential for phase‐dependent TMS.

Sensorimotor mu‐rhythm phase modulates corticospinal excitability; however, the influence of mu‐phase varies across individuals and could be related to microstructural connectivity between primary somatosensory (S1) and motor (M1) cortices. Our findings suggest that higher S1–M1 microstructural connectivity is associated with a stronger mu‐phase effect on corticospinal excitability.

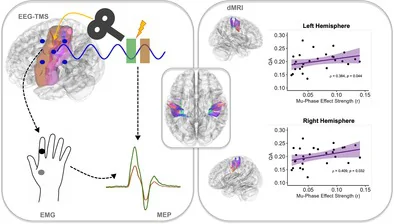

## 1 Introduction

The employment of brain‐state dependent stimulation has been favored in recent years to reduce the variability in effects and increase the benefits of non‐invasive brain stimulation methods, such as transcranial magnetic stimulation (TMS) (Baur et al. 2020; Bergmann 2018; Wischnewski et al. 2022; Zrenner et al. 2018, 2023). Brain state is generally determined through electroencephalography (EEG) oscillations (Wischnewski et al. 2022; Zrenner et al. 2018, 2023). In particular, the sensorimotor mu‐rhythm phase reflects fluctuating corticospinal excitability states. When TMS is delivered to the primary motor cortex (M1) at the trough or early rising phase of the mu‐rhythm, larger motor‐evoked potentials (MEP) are elicited, representing a high corticospinal excitability state (Suresh and Hussain 2023; Wischnewski et al. 2022; Zrenner et al. 2018, 2023). The opposite (i.e., smaller MEPs) is found when delivering TMS at the mu‐phase peak, thus indicating low excitability (Suresh and Hussain 2023; Wischnewski et al. 2022; Zrenner et al. 2018, 2023). Although multiple studies have shown an effect of mu‐phase on MEP amplitude, this mu‐phase effect is variable at the single‐subject level (Hougland et al. 2025; Kirchhoff et al. 2024; Zrenner et al. 2023). In fact, only approximately half of subjects demonstrate a significant mu‐phase effect (Hougland et al. 2025; Zrenner et al. 2023), and some studies found no effect of mu‐phase on corticospinal excitability (Madsen et al. 2019).

The variability in the strength of the mu‐phase effect could be, in part, due to structural differences in the brain. The effects and propagation of TMS are influenced by white matter architecture and microstructure (Martin‐Signes et al. 2024; Ning et al. 2022; Rodriguez‐Herreros et al. 2015), which can be assessed with diffusion magnetic resonance imaging (dMRI). For example, fractional anisotropy (FA) is a dMRI measure of the microstructural integrity of white matter tissue. Lower FA in cerebral white matter, pyramidal tract, and corpus callosum has been associated with higher resting motor thresholds (RMT) (Kloppel et al. 2008). Additionally, FA of corpus callosum fibers was positively correlated with the level of M1 interhemispheric inhibition measured with TMS (Koerte et al. 2009; Wahl et al. 2007). In regard to mu‐rhythm phase, higher fiber density and cross‐section of callosal motor fibers were positively related to the synchronization of mu‐rhythm peak and trough phases between the left and right M1 (Momi et al. 2022).

Previous research has shown that the phase of the mu‐rhythm signal extracted from the primary somatosensory cortex (S1) is more related to corticospinal excitability states than that from M1, even though TMS is delivered to M1 (Zrenner et al. 2022). It is hypothesized that specific neuronal pathways from S1 to M1 account for this relationship (Zrenner et al. 2022). If this is the case, it would be expected that alterations in the structural integrity and connectivity of the S1–M1 pathway would impact the strength of the mu‐rhythm effect on corticospinal excitability, tested by MEP amplitude. dMRI analysis of S1–M1 connectivity could provide mechanistic evidence of physiological inputs influencing mu‐phase modulation of corticospinal excitability. Additionally, if structural properties of S1–M1 fibers dictate the influence of mu‐rhythm phase on MEP amplitude, researchers and clinicians could use dMRI to predict an individual's potential for effective phase‐dependent stimulation.

In this study, we examined the relationship between dMRI measures of white matter microstructure of the left and right S1–M1 U‐shaped fibers and the mu‐rhythm phase effect strength in healthy adults. We used generalized q‐sampling imaging (GQI) (Yeh et al. 2010), a deterministic and model‐free fiber tracking approach, and neurite orientation dispersion and density imaging (NODDI) (Zhang et al. 2012) to quantify properties of white matter microstructure. Additionally, we used whole‐brain correlational tractography to explore other regions potentially associated with the mu‐rhythm phase effect. We applied TMS to the left M1 to assess the effect of mu‐phase on corticospinal excitability of the right hand.

## 2 Methods

### 2.1 Participants and Study Design

The EEG–TMS data were previously reported in Hougland et al. (2025). A total of 28 right‐handed participants (15 males, 13 females, mean age: 26.3 ± 4.5 year; range: 21–36 year) completed two sessions: an MRI acquisition and an EEG–TMS experiment. Participants with a history of neurological disease or substance use, or any contraindications to TMS or MRI were excluded (Rossi et al. 2021). All participants gave written informed consent. Studies were approved by the Ethics Committee of the Medical Faculty at the University of Tübingen (Protocol numbers: 716/2014BO2 and 810/2021BO2) and conducted in accordance with the Declaration of Helsinki (2013 revision).

### 2.2 MRI Acquisition

MRIs were obtained during a single session with a 3 T Siemens Prisma system and a 64‐channel head coil. Diffusion‐weighted images were obtained using a multi‐shell scheme (b‐values = 900, 1600, and 2500 s/mm^2^), diffusion sampling directions of 18, 32, and 50 (respectively), in‐plane resolution and slice thickness of 1.5 mm, repetition time (TR) of 5800 ms, and echo time (TE) of 107 ms. A reverse phase‐encoding scan was acquired for susceptibility artifact and current distortion correction (b‐values = 0 and 2500 s/mm^2^, in‐plane resolution and slice thickness of 1.5 mm, TR of 5800 ms, TE of 107 ms, and 7 volumes).

### 2.3 EEG–TMS Acquisition

Open‐loop (i.e., brain‐state independent) single TMS pulses (800 in 22 participants, 1200 in three blocks of 400 in 6 participants) were applied to the left M1 hand hotspot at 110% of RMT. EMG was recorded from the right first dorsal interosseous (FDI) and abductor pollicis brevis (APB) muscles. EEG was measured with TMS‐compatible caps (22 participants: 64‐channel; 6 participants: 128‐channel; EasyCap, Germany). Signals were sampled at 5 kHz and amplified using a 24‐bit biosignal amplifier (NeurOne, Bittium, Finland). TMS was delivered with a MagVenture X100 stimulator and a Cool‐B35 (*N* = 22) or Cool‐B65 SLS coil (*N* = 6) and an interstimulus interval (ISI) of 2.25 ± 0.05 s (*N* = 22) or 2.5–3.5 s (*N* = 6). For hotspot determination, the coil was placed at an approximately 45‐degree angle to the longitudinal fissure to induce for the second phase of the biphasic stimulus waveform a posterolateral‐anteromedial current in the brain. Coil position and orientation was adjusted until the position where the largest and most consistent MEPs were elicited. RMT was defined as the intensity needed to produce an MEP of at least 50 μV in 50% of trials (Rossini et al. 2015). A neuronavigation system (Localite, Germany) was used throughout the experiment to track coil position and orientation. During the TMS protocol, participants were seated in a relaxed position with their head position secured using a vacuum pillow (B.u.W Schmidt GmbH, Germany) and instructed to focus on a fixation cross.

*Diffusion MRI preprocessing*. Diffusion MRIs were preprocessed using DSI Studio (version “Hou”, http://dsi‐studio.labsolver.org) (Yeh 2025). Image quality was verified following DSI Studio's quality control procedure (Yeh, Panesar, et al. 2019; Yeh, Zaydan, et al. 2019). The images were corrected for subject motion, eddy currents, and susceptibility‐induced distortions using FSL's topup/eddy (Andersson et al. 2003; Smith et al. 2004). The restricted diffusion was quantified using restricted diffusion imaging (Yeh et al. 2017) and data were reconstructed using GQI (diffusion sampling length ratio = 1.25) (Yeh et al. 2010). GQI is a model‐free method for resolving fiber orientations that performs better than diffusion tensor imaging (DTI) at quantifying kissing and crossing fibers.

**FIGURE 1:**
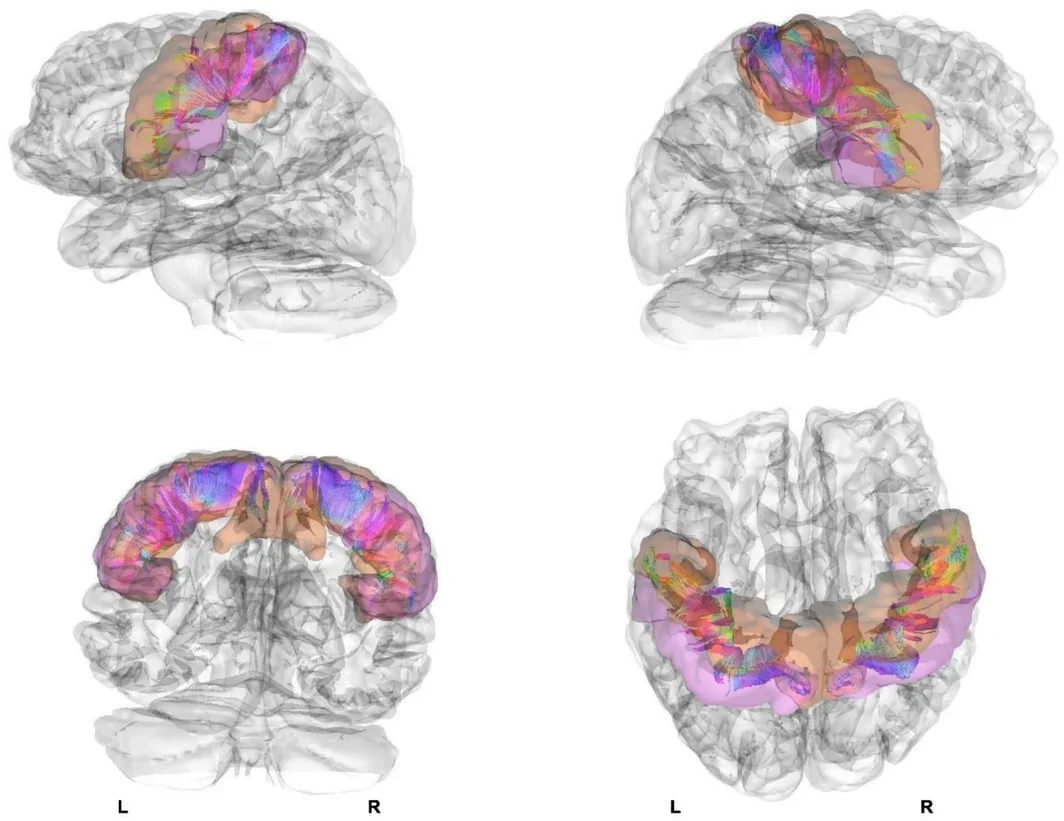
Primary somatosensory cortex to primary motor cortex (S1–M1) U‐shaped fiber tractography. Illustrated is one representative participant's fiber tractography results for the S1–M1 connecting fibers. Four views are displayed: Left lateral (top left), right lateral (top right), caudal coronal (bottom left), and superior axial (bottom right). The U‐shaped fibers can be seen connecting the precentral (orange overlay) and postcentral (pink overlay) gyri. The fiber colors represent the direction of the fibers (red: Left–right, green: Anterior–posterior, blue: Superior–inferior). L and R indicate the left and right hemispheres, respectively.

*Tractography*. S1–M1 U‐shaped fiber tractography was performed by assigning the M1 and S1 as regions of interest (ROIs). The ROIs were selected using the Brainnetome atlas (M1: PrG_6_1‐6 & PCL_2_1‐2; S1: PoG_4_1‐4) (Fan et al. 2016). The S1 was set as a seed region and the M1 as an end region. Tractography was performed separately for both the left and right hemispheres. The following tracking parameters were used: angular threshold of 45 degrees, step size of 0.5 mm, minimum length of 20 mm, maximum length of 200 mm, 100,000 seeds, and 4 iterations of automatic topology‐informed pruning (Yeh, Panesar, et al. 2019; Yeh, Zaydan, et al. 2019). Tracks were manually pruned as needed by the same investigator (JRH). An example participant's tractography is shown in Figure 1.

*Microstructural properties*. Quantitative anisotropy (QA) and DTI measures, such as FA, radial diffusivity (RD), and axial diffusivity (AD), were extracted from the tracts. QA is associated with axonal density, while FA is nonspecifically associated with axonal integrity and is more sensitive to edema, inflammation, and demyelination than QA (Yeh et al. 2013; Yeh, Panesar, et al. 2019; Yeh, Zaydan, et al. 2019). RD is related to myelination (increases with decreasing myelination) (Song et al. 2005), and AD is nonspecifically associated with axonal density (increases with increasing axonal density) (Budde et al. 2009). The preprocessed dMRIs and binary brain masks (created with MRtrix3's dwi2mask) were used for the NODDI analysis (Zhang et al. 2012). NODDI metrics, including orientation distribution index (ODI), neurite density index (NDI), and isotropic fraction (F_iso_), were extracted for the S1–M1 tracts.

*Correlational tractography*. Additionally, whole‐brain correlational tractography was performed to explore other regions where QA and FA are potentially associated with the mu‐phase effect strength (Yeh et al. 2021). A database was created based on the GQI reconstruction of each participant's dMRI. Non‐parametric Spearman partial correlations were used to correlate the mu‐phase effect strength and QA/FA. QA values were normalized. A T‐value threshold of 2.0 and a length threshold of 20 voxels were used. A total of 8 iterations of topology‐informed pruning were done to filter tracks. A total of 4000 randomized permutations were applied to estimate the false discovery rate (FDR). Statistical significance was set at FDR < 0.05. Tracts were identified using DSI Studio's “Recognize and Cluster” function. Tracts with fewer than 50 fibers were excluded as spurious fibers.

*EMG/EEG preprocessing*. EMG and EEG data were preprocessed and analyzed using MATLAB (v2023a, MathWorks, Natick, MA, USA), EEGLAB (v2024.2), and R (v4.2.2, R Foundation for Statistical Computing, Vienna, Austria). EMG data were epoched for 200 ms around the TMS pulse, downsampled to 1 kHz, baseline corrected, and linearly detrended. A 50 Hz notch filter was applied to the baseline. Peak‐to‐peak MEP amplitudes of the FDI (*N* = 26) or APB (*N* = 2) were extracted for each trial from 20 to 40 ms post‐TMS and natural log‐transformed. Exclusion of trials in the final analysis included pre‐innervation exceeding an amplitude of 50 μV in the pre‐stimulation period or MEP outliers based on the 1.5 interquartile range rule (values below Q1 − 1.5 × IQR or above Q3 + 1.5 × IQR are considered outliers, where IQR = Q3 − Q1) (Tukey 1977).

EEG data were processed with a C3‐Hjorth filter (64 channel cap adjacent electrodes: FC5, FC1, CP5, and CP1; 128 channel cap adjacent electrodes: FCC5h, FCC3h, CCP5h, and CCP3h) applied to the data to obtain mu‐phase estimates at the time of TMS delivery (Hjorth 1991; Zrenner et al. 2023). For each trial, data were epoched from −705 to −5 ms, baseline‐corrected, detrended, and downsampled to 1 kHz. Trials were excluded if any channel in the C3‐Hjorth montage exceeded the 150 μV range (Hougland et al. 2025; Kirchhoff et al. 2024; Zrenner et al. 2023). Data were bandpass filtered at 9–13 Hz using a Hamming window using FIR filter and mu‐rhythm phase at TMS delivery for the left hemisphere was estimated with the PHASTIMATE toolbox (filter order = 128, edge samples removed = 64, autoregressive model order = 30) (Zrenner et al. 2020).

### 2.4 Statistical Analysis

Analyses for S1‐M1 U‐shaped fibers were performed in R (v4.2.2) with significance at *p* < 0.05. Even mu‐phase trial distribution was confirmed with a Rayleigh test of uniformity. A circular‐to‐linear correlation (‘Directional’ package, v7.1) determined participants' mu‐phase effect on MEP amplitude. The correlation coefficient (*r*) was used as a measure of the strength of the mu‐phase effect (a higher *r* reflecting a stronger mu‐phase effect on MEP amplitude). Spearman's rank correlations were used to assess relationships between dMRI measures (QA, FA, RD, AD, ODI, NDI, F_iso_) and the mu‐phase effect strength (*r*) within S1–M1 U‐shaped fibers. These correlations were not corrected for multiple comparisons, given a strong a priori, biologically motivated hypothesis regarding the functional relevance of S1–M1 connectivity (Zrenner et al. 2022). For the exploratory, whole‐brain correlational tractography, *p* values were FDR‐corrected.

## 3 Results

### 3.1 Mu‐Phase Effect Strength

The mean ± standard deviation of the mu‐phase effect strength across all participants was 0.065 ± 0.037 and ranged from 0.021 to 0.141.

### 3.2 S1–M1 U‐Shaped Fibers

**FIGURE 2:**
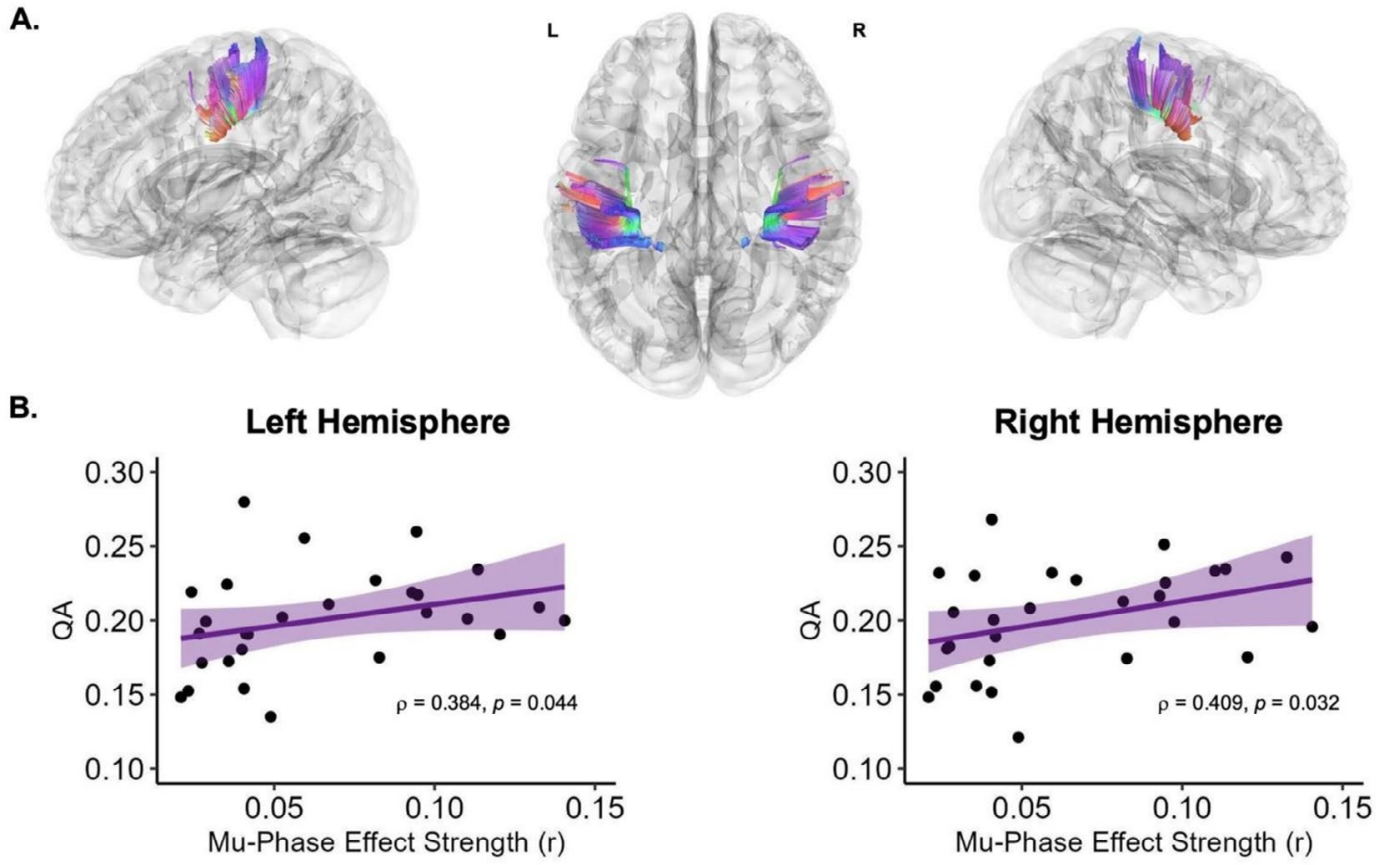
Correlation between mu‐phase effect strength and quantitative anisotropy of S1–M1 U‐shaped fibers. Shown is the group (*N* = 28) averaged S1–M1 U‐shape fiber tractography (A; from left to right: Right‐lateral view, top‐axial view, and left‐lateral view) and plots (B) illustrating the correlation between mu‐phase effect strength (r) and quantitative anisotropy (QA). Significant positive correlations were found between both left (*ρ* = 0.384, *p* = 0.044) and right (*ρ* = 0.409, *p* = 0.032) hemisphere S1–M1 U‐shaped fiber QAs and mu‐phase effect strength on MEP amplitudes. Correlations are Spearman's rank correlations. In A, the fiber colors represent the direction of the fibers (red: Left–right, green: Anterior–posterior, blue: Superior–inferior). In B, each black point represents an individual subject, the purple lines represent the linear model fit, and the purple shadings show the 95% confidence intervals.

The mu‐phase effect strength was positively correlated with QA of the S1–M1 U‐shaped fibers in both the left (*ρ* = 0.384, *p* = 0.044) and right (*ρ* = 0.409, *p* = 0.032) hemispheres (Figure 2). No significant correlations were found between the mu‐phase effect strength and FA, RD, or AD in either hemisphere (all *p* > 0.05). Furthermore, NODDI metrics (ODI, NDI, F_iso_) were not significantly correlated with mu‐phase effect strength of the S1–M1 U‐shaped fibers (all *p* > 0.05). Table 1 shows all metric correlations.

**TABLE 1:** Spearman's rank correlations between S1–M1 U‐shaped fiber metrics and mu‐phase effect strength.

Metric	Left hemisphere	Right hemisphere
Quantitative Anisotropy	0.384*	0.409*
Fractional Anisotropy	0.273	0.267
Radial Diffusivity	−0.236	−0.336
Axial Diffusivity	0.115	0.033
Orientation Distribution Index	0.314	0.242
Neurite Density Index	−0.245	−0.065
Isotropic Fraction	0.241	0.088

* *p* < 0.05.

### 3.3 Whole‐Brain Tractography

**FIGURE 3:**
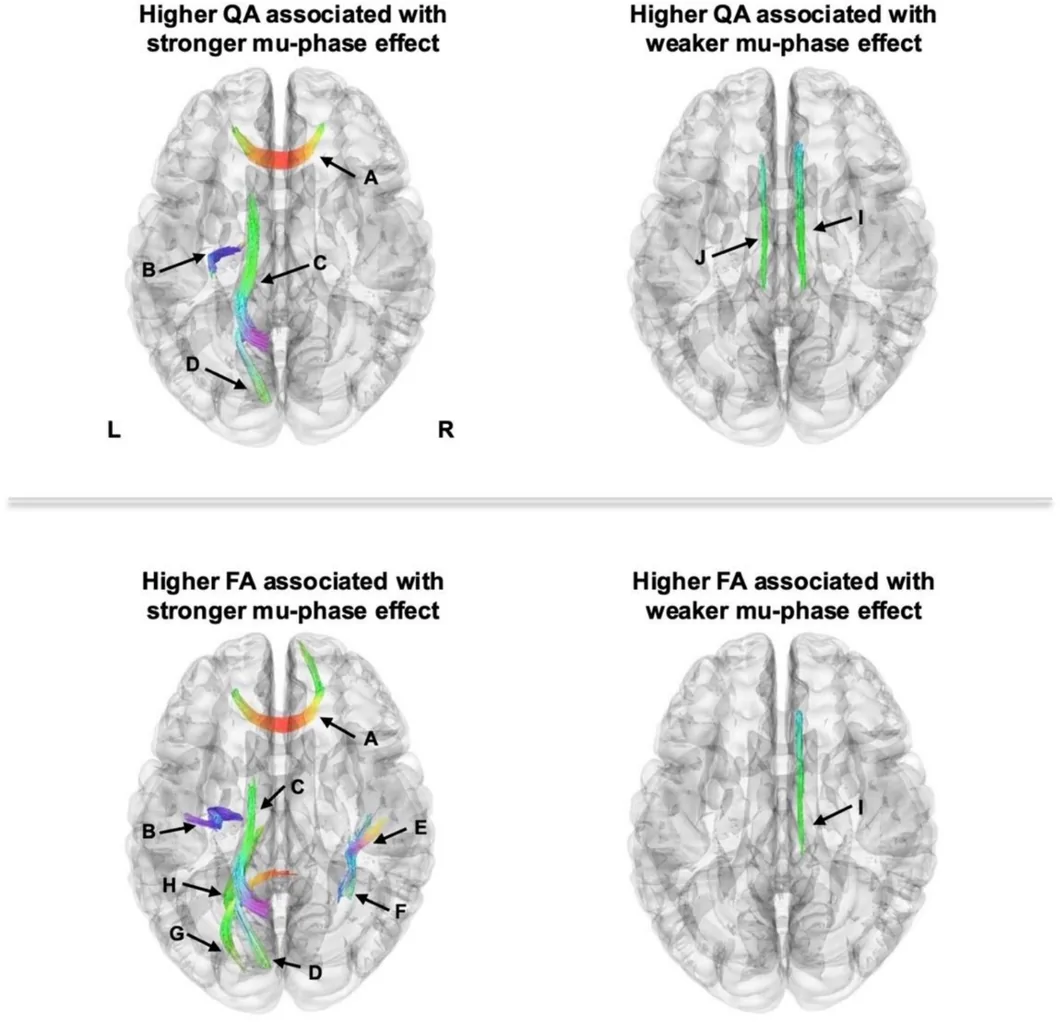
Whole‐brain correlational tractography of quantitative and fractional anisotropy and the mu‐phase effect strength. Mu‐phase effect strength on MEP amplitudes showed significant positive correlations (corrected *p* < 0.001) with quantitative (QA, top left) and fractional anisotropy (FA, bottom left) in the forceps minor (A), left corticospinal tract (B), left superior longitudinal fasciculus I (C), and left parahippocampal parietal (D) fibers. Positive correlations (corrected *p* < 0.001) were also found with FA in the right middle longitudinal fasciculus (E), tapetum (F), forceps major (G), and middle cerebellar peduncle (H) fibers (bottom left). In the right hemispheric cingulum parolfactory fibers (I) QA (corrected *p* < 0.001, top right) and FA (−corrected *p* = 0.001, bottom right) were negatively correlated with the mu‐phase effect strength. QA (top right) of the left hemisphere cingulum frontal parahippocampal fibers (J) was also negatively correlated (corrected *p* < 0.001) with the mu‐phase effect strength.

In an exploratory, whole‐brain, correlational tractography analysis, QA and FA of several pathways were positively correlated (FDR‐corrected *p* < 0.001) with the mu‐phase effect strength, including the forceps minor, left corticospinal tract, left superior longitudinal fasciculus I, and left parahippocampal parietal fibers (Figure 3). Additionally, positive correlations were found for FA in the right middle longitudinal fasciculus, tapetum, forceps major, and middle cerebellar peduncle fibers (corrected *p* < 0.001). Negative correlations were found between mu‐phase effect strength and QA/FA in the right cingulum parolfactory fibers (QA corrected *p* < 0.001; FA corrected *p* = 0.001), as well as for QA in the left cingulum frontal parahippocampal fibers (corrected *p* < 0.001).

## 4 Discussion

In this study, we investigated the relationship between the structural connectivity between ipsilateral S1 and M1 and the strength of the influence of pre‐stimulus sensorimotor mu‐rhythm phase on MEP amplitudes. Open‐loop TMS was delivered to the left M1 hand hotspot, and the mu‐phase effect strength was quantified as the correlation coefficient from a circular‐to‐linear correlation of mu‐phase and MEP amplitude. Tractography was performed to evaluate the U‐shaped fibers connecting S1 and M1, and dMRI microstructural measures, such as QA, FA, RD, and AD, as well as NODDI metrics (ODI, NDI, F_iso_), were calculated and correlated with the mu‐phase effect strength. We found that higher QA of the S1–M1 U‐shaped fibers was associated with a stronger effect of mu‐phase on MEP amplitudes. No significant relationships were found for the other dMRI and NODDI metrics. In addition, whole‐brain correlational tractography identified fiber tracts in other regions associated with the mu‐phase effect.

Over the past several years, multiple studies across different laboratories have established that mu‐phase modulates corticospinal excitability (Baur et al. 2020; Bergmann et al. 2019; Ozdemir et al. 2022; Suresh and Hussain 2023; Wischnewski et al. 2022; Zrenner et al. 2018, 2023). Typically, the mu‐rhythm is extracted from the C3‐Hjorth Laplacian filtered EEG signal (Wischnewski et al. 2022; Zrenner et al. 2022, 2018, 2023). The C3 electrode lies over the postcentral gyrus, corresponding to the region of S1. The TMS hotspot for eliciting MEPs from the hand muscles is generally found in the precentral gyrus “hand knob”, typically an omega shaped landmark that identifies the M1 hand representation area (Yousry et al. 1997). Although the TMS hotspot is generally 2–3 cm anterior to the C3 electrode (Zrenner et al. 2022), extracting the EEG signal from an electrode/montage closer to M1, or the TMS hotspot, negates the mu‐phase effect on corticospinal excitability (Karabanov et al. 2021; Madsen et al. 2019; Zrenner et al. 2022). Surprisingly, Zrenner et al. 2022 found that this is the case even though the mean phase difference between the C3‐Hjorth and FCC3h‐Hjorth (M1 region) signals was insignificant (Zrenner et al. 2022). However, oscillatory phase estimates between C3‐Hjorth and FCC3h‐Hjorth disagreed in the majority of trials. Collectively, these findings suggest that EEG signals extracted from S1 play a significant role in corticospinal output responses from M1 stimulation.

In the current study, our results support and extend prior work by providing complementary evidence of the relevance of S1–M1 interactions for phase‐dependent TMS. While previous studies have demonstrated the S1–M1 interaction using EEG (Zrenner et al. 2022), our use of dMRI offers greater spatial resolution to characterize intrahemispheric connectivity. Higher QA of the S1–M1 U‐shaped fibers, in both the stimulated (left) and non‐stimulated (right) hemispheres, was associated with a stronger mu‐phase effect on MEP amplitudes. QA measures the density of anisotropic diffusion water in a fiber bundle; it increases with increasing axonal density and organization (Yeh, Panesar, et al. 2019; Yeh, Zaydan, et al. 2019). These results suggest that the microstructural organization of the connecting fibers between S1 and M1 may facilitate the effect of mu‐phase on MEPs.

One reason for this could be that a higher density of connections between S1 and M1 leads to improved communication and synchronization between the two nodes. The improved communication between S1 and M1 may result in better phase‐locking. If the phases in S1 and M1 are more synchronous, then the effect of the S1–M1 combined sensorimotor mu‐phase on MEP amplitudes is stronger. There is evidence that greater interhemispheric phase‐locking is associated with higher corticospinal excitability states (Marzetti et al. 2024; Vetter et al. 2023). Moreover, higher fiber density and cross‐section of fibers connecting left and right M1 have been shown to improve the phase‐locking between the two nodes (Momi et al. 2022). Our results indicate that this principle may apply to intrahemispheric connections as well. More mechanistically, the trough of the C3‐Hjorth sensorimotor mu‐rhythm corresponds to scalp‐to‐cortex inward currents, resulting in depolarization of the apical dendrites of radially oriented principal cells in S1. Their increased activity at that time will result in increased excitatory synaptic input in M1 corticospinal cells a few milliseconds later, making them more excitable to TMS (Laakso et al. 2014; Zrenner et al. 2023).

Future studies could examine whether functional connectivity measures (e.g., phase‐locking, resting‐state functional MRI, paired‐pulse TMS measures) between S1 and M1 are correlated with S1–M1 structural connectivity and whether they provide additional information to better explain the interindividual differences in the effect of mu‐phase on corticospinal excitability.

Although QA was significantly correlated with the mu‐phase effect strength, other dMRI and NODDI metrics were not. This is likely due to differences in diffusion modeling methods and the biological basis of the metrics (Beaulieu 2002; Yeh et al. 2021). QA has been shown to be less sensitive to partial volume effects and edema, and QA‐aided tractography outperforms FA‐aided tractography (Yeh et al. 2013). Notably, QA‐aided tractography is better at resolving crossing fibers, which is relevant for the S1–M1 U‐shaped fibers, which cross with the corticospinal tract and callosal fibers. The DTI metrics (FA, RD, and AD) may therefore be less sensitive for elucidating relationships between S1–M1 U‐shaped fibers and the mu‐phase effect. Similarly, NODDI uses a different computational method to model dMRI. While NODDI may be optimal for exploring tissue composition and properties (Zhang et al. 2012), GQI (used to assess QA) may be advantageous for mapping white matter connectivity, thus making it more relevant for investigating S1–M1 connectivity instead of microstructure.

There is a limited number of studies exploring S1–M1 connections with dMRI due to challenges in resolving U‐shaped tracts. However, the structure–function relationship of S1 and M1 has been investigated previously (Pron et al. 2021; Thompson et al. 2017). In Thompson et al. (2017), FA, perpendicular diffusivity, and mean diffusivity of the hand S1–M1 U‐shaped fibers were correlated with performance on a pegboard test for manual dexterity (Thompson et al. 2017). Furthermore, the position of three bundles of hand‐related U‐shaped fibers was associated with the handedness index of participants (Pron et al. 2021). Thus, S1–M1 structural and functional connectivity are complementarily related.

S1 and M1 are believed to communicate through both direct and indirect pathways (Catani et al. 2012; Davis et al. 2022; Thompson et al. 2017). Direct cortico‐cortical pathways between S1 and M1 have been studied using paired‐pulse TMS, in which a conditioning TMS pulse is delivered to S1 with a specific ISI prior to a test pulse to M1 in order to measure the interaction between the two nodes (Brown et al. 2019; Davis et al. 2022). Both studies found an inhibitory effect of S1 conditioning stimulation on MEP amplitude elicited by M1 test stimulation at a very short ISI of 1 ms but not at a longer ISI of 2.5 ms. While the mechanism of this MEP inhibition has not been fully elucidated, it may be best explained by refractoriness of S1–M1 U‐shaped fibers that are excited by the conditioning and test stimulus, proving the relevance of S1 excitatory input to corticospinal neurons in M1. Furthermore, whole‐cell recordings have suggested that S1 and M1 interact via direct monosynaptic connections and that S1 input produces large postsynaptic responses indicating a sensorimotor feedforward system (Rocco‐Donovan et al. 2011). Additionally, S1 has been shown to be a driver to pyramidal cells of M1 through a paired‐pulse depression mechanism (Petrof et al. 2015), again suggesting that M1 output is largely influenced by direct communication with S1.

There is evidence that interactions between S1 and M1 also occur through polysynaptic thalamo‐cortical pathways (Arce‐McShane et al. 2016; Catani et al. 2012; Davis et al. 2022; Thompson et al. 2017). M1 receives somatosensory input directly from the thalamus during movement execution (Takahashi et al. 2021). However, S1 can act as a bridge between the thalamus and M1 to relay somatosensory input. For example, pharmacological inactivation of S1 neurons in a monkey disrupts its precision grasping performance (Hikosaka et al. 1985). Moreover, when tetanic stimulation is applied to S1 in monkeys, long‐term potentiation (LTP) effects are seen in M1, but not when tetanic stimulation is applied to the thalamus (Iriki et al. 1991; Sakamoto et al. 1987). Catani et al. (2012) suggest that the S1–M1 U‐shaped fibers represent the terminal connections of the indirect somatosensory thalamo‐cortical pathway to M1.

We also examined other tracts that are associated with the mu‐phase effect on corticospinal excitability by performing exploratory whole‐brain correlational tractography. Multiple tracts were found to be significantly positively correlated with the mu‐phase effect strength, including callosal, corticospinal, longitudinal fasciculus, and parahippocampal parietal tracts. Several of these tracts are plausibly relevant to the propagation of TMS effects. The corticospinal tract is the pathway that mediates the activation of corticospinal neurons by TMS to spinal cord and muscle (Groppa et al. 2012). Properties of the corticospinal tract have been related to corticospinal excitability. Decreases in FA of the corticospinal tract are associated with increases in RMT (Kloppel et al. 2008). Additionally, greater corticospinal excitability is associated with larger corticospinal tract volume in participants with anterior cruciate ligament reconstruction (Lepley et al. 2020). We found that higher QA and FA of the corticospinal tract were reflective of a stronger effect of mu‐phase on MEP amplitudes. These results may indicate that participants with higher corticospinal tract integrity may also show higher sensitivity to the effects of sensorimotor mu‐phase. A recent study found that the mu‐phase effect on MEPs is intact in stroke patients, with mu‐phase dependent modulation of MEPs observed in the contralesional and ipsilesional hemispheres (Wischnewski et al. 2025). However, the mu‐phase effect strength was weaker in stroke patients with stronger upper extremity motor impairment, which is heavily influenced by corticospinal tract injury (Feng et al. 2015; Lin et al. 2019; Wischnewski et al. 2025). These findings further support that corticospinal tract integrity is relevant for mu‐phase dependent modulation of corticospinal excitability. Thus, in addition to S1–M1 connectivity, corticospinal tract integrity should be considered as a predictor for the effectiveness of phase‐dependent TMS, especially in individuals with corticospinal tract injury, such as in stroke patients.

We found the positive correlation of QA and FA with mu‐phase effect strength in the superior longitudinal fasciculus I tract as well. The superior longitudinal fasciculus I connects the parietal and frontal lobes and is important for the integration of sensorimotor information, which may make it an important tract for the mu‐rhythm (Vergani et al. 2021). Unexpectedly, non‐motor related callosal regions were associated with the mu‐phase effect strength. Why these non‐motor callosal and other identified tracts (parahippocampal parietal and right middle longitudinal fasciculus) are reflective of mu‐phase effect strength on corticospinal excitability needs to be further studied. Similarly, we found that fibers within the cingulum tract were negatively correlated with mu‐phase effect strength, but it is not clear what physiological mechanisms contribute to the influence of cingulum tract fibers on sensorimotor mu‐rhythm.

In this study, we focused on the effect of mu‐rhythm phase on corticospinal excitability (as measured via MEP amplitudes) and its relationship to S1–M1 connectivity. However, mu‐rhythm phase also influences TMS‐evoked potentials (TEP) from EEG. The trough and falling phases of the mu‐rhythm are associated with larger TEP amplitudes (Desideri et al. 2019; Perera et al. 2024). Our results indicate that S1–M1 structural connectivity is relevant for the modulatory effect of mu‐phase. This could be further validated by examining to what extent S1–M1 connectivity is also correlated with the mu‐phase effect strength on TEP amplitudes reflecting cortical excitability.

This study has a few limitations. First, the reconstruction of S1–M1 U‐shaped fibers is difficult, and few studies have explored these connections (Pron et al. 2021). Furthermore, the few studies that have investigated S1–M1 tracts use different dMRI models and methods for fiber tractography, which makes comparability between studies challenging (Catani et al. 2012; Pron et al. 2021; Thompson et al. 2017). Additionally, there is potential for bias with the manual pruning of the S1–M1 tracts, although we attempted to standardize this by having one investigator perform all manual pruning. Second, we did not standardize time between the MRI and TMS sessions. Although we do not expect major changes to occur in structural connectivity in the absence of injury or disease, structural plasticity can adapt rapidly in response to changes in behavior or learning and thus cannot be entirely ruled out (Hofstetter and Assaf 2017; Villemonteix et al. 2023), but such an effect would only underestimate the reported findings. Third, the circular‐to‐linear correlation to assess the mu‐phase effect assumes a sinusoidal relationship between mu‐rhythm phase and MEP amplitude. However, this may be an inaccurate model for the mu‐rhythm considering the evidence that the trough phase is shorter than the peak (Bender et al. 2023; Bergmann et al. 2019; Ross et al. 2022; Schaworonkow and Nikulin 2019; Zrenner et al. 2023). Lastly, the two subgroups of data used in the analyses have several protocol differences (number of trials, interstimulus interval, TMS coil, and number of EEG channels), although we do not expect any significant contributions of these minor differences to the results.

## 5 Conclusions

Structural connectivity between S1 and M1 influences the strength of sensorimotor mu‐phase on MEP amplitudes elicited from TMS to M1. Other white matter tracts could also be relevant to the mu‐phase effect strength. The results of this study provide evidence that structural connections between S1 and M1, reflective of direct communication between the two nodes, are linked to the efficacy of phase‐dependent TMS in healthy adults.

## Author Contributions

**Juliana R. Hougland:** conceptualization, formal analysis, visualization, writing – original draft, writing – review and editing. **Timo Roine:** supervision, writing – review and editing. **Andreas Jooß:** supervision, writing – review and editing. **Dania Humaidan:** supervision, writing – review and editing. **Ulf Ziemann:** funding acquisition, supervision, writing – review and editing.

## Funding

This work was supported by H2020 European Research Council, 810377; Deutsche Forschungsgemeinschaft, 265732651; Finnish Brain Foundation.

## Conflicts of Interest

The authors declare no conflicts of interest.

## Data Availability

The data that support the findings of this study are available on request from the corresponding author. The data are not publicly available due to privacy or ethical restrictions.
